# Influenza-Associated Excess Mortality by Age, Sex, and Subtype/Lineage: Population-Based Time-Series Study With a Distributed-Lag Nonlinear Model

**DOI:** 10.2196/42530

**Published:** 2023-01-11

**Authors:** Li Li, Ze-Lin Yan, Lei Luo, Wenhui Liu, Zhou Yang, Chen Shi, Bo-Wen Ming, Jun Yang, Peihua Cao, Chun-Quan Ou

**Affiliations:** 1 State Key Laboratory of Organ Failure Research, Department of Biostatistics School of Public Health Southern Medical University Guangzhou China; 2 Guangzhou Center for Disease Control and Prevention Guangzhou China; 3 School of Public Health Guanghzou Medical University Guangzhou China; 4 Clinical Research Center Zhujiang Hospital Southern Medical University Guangzhou China

**Keywords:** influenza, disease burden, distributed-lag nonlinear model, excess mortality, harvesting effects

## Abstract

**Background:**

Accurate estimation of the influenza death burden is of great significance for influenza prevention and control. However, few studies have considered the short-term harvesting effects of influenza on mortality when estimating influenza-associated excess deaths by cause of death, age, sex, and subtype/lineage.

**Objective:**

This study aimed to estimate the cause-, age-, and sex-specific excess mortality associated with influenza and its subtypes and lineages in Guangzhou from 2015 to 2018.

**Methods:**

Distributed-lag nonlinear models were fitted to estimate the excess mortality related to influenza subtypes or lineages for different causes of death, age groups, and sex based on daily time-series data for mortality, influenza, and meteorological factors.

**Results:**

A total of 199,777 death certificates were included in the study. The average annual influenza-associated excess mortality rate (EMR) was 25.06 (95% empirical CI [eCI] 19.85-30.16) per 100,000 persons; 7142 of 8791 (81.2%) deaths were due to respiratory or cardiovascular mortality (EMR 20.36, 95% eCI 16.75-23.74). Excess respiratory and cardiovascular deaths in people aged 60 to 79 years and those aged ≥80 years accounted for 32.9% (2346/7142) and 63.7% (4549/7142) of deaths, respectively. The male to female ratio (MFR) of excess death from respiratory diseases was 1.34 (95% CI 1.17-1.54), while the MFR for excess death from cardiovascular disease was 0.72 (95% CI 0.63-0.82). The average annual excess respiratory and cardiovascular mortality rates attributed to influenza A (H3N2), B/Yamagata, B/Victoria, and A (H1N1) were 8.47 (95% eCI 6.60-10.30), 5.81 (95% eCI 3.35-8.25), 3.68 (95% eCI 0.81-6.49), and 2.83 (95% eCI –1.26 to 6.71), respectively. Among these influenza subtypes/lineages, A (H3N2) had the highest excess respiratory and cardiovascular mortality rates for people aged 60 to 79 years (20.22, 95% eCI 14.56-25.63) and ≥80 years (180.15, 95% eCI 130.75-227.38), while younger people were more affected by A (H1N1), with an EMR of 1.29 (95% eCI 0.07-2.32). The mortality displacement of influenza A (H1N1), A (H3N2), and B/Yamagata was 2 to 5 days, but 5 to 13 days for B/Victoria.

**Conclusions:**

Influenza was associated with substantial mortality in Guangzhou, occurring predominantly in the elderly, even after considering mortality displacement. The mortality burden of influenza B, particularly B/Yamagata, cannot be ignored. Contrasting sex differences were found in influenza-associated excess mortality from respiratory diseases and from cardiovascular diseases; the underlying mechanisms need to be investigated in future studies. Our findings can help us better understand the magnitude and time-course of the effect of influenza on mortality and inform targeted interventions for mitigating the influenza mortality burden, such as immunizations with quadrivalent vaccines (especially for older people), behavioral campaigns, and treatment strategies.

## Introduction

Seasonal influenza has been associated with a large number of deaths, both in China and globally [1-5]. An accurate gauge of the mortality burden of the seasonal influenza epidemic is of great importance for understanding the impact of influenza, as well as for formulating and adjusting corresponding prevention and control measures [6]. This has been challenging, as laboratory diagnoses of influenza are not routinely made, and it is difficult to distinguish influenza virus infections from other respiratory pathogen infections based on nonspecific clinical symptoms [7]. Additionally, older adults are more likely to die from influenza-triggered complications, but influenza is seldom recorded as the cause of death in the mortality registration system [8]. Therefore, laboratory-confirmed deaths are an underestimate of the mortality burden of influenza [6].

Many studies have assessed the mortality burden of influenza with statistical models [3,6-19]. There have been substantial variations in estimates of influenza-associated excess mortality and in analytical strategies, including the definition of the lag period between influenza activity and mortality [20]. Previous studies have assumed that the lag was zero weeks, months, or years or one week; correspondingly, a simple influenza activity proxy was often included in the model, that is, a proxy lag of 1 week was used [20]. Lytras et al [21] examined the delayed effect of influenza activity on all-cause mortality using a distributed-lag nonlinear model (DLNM), allowing a flexible lag-response relationship between influenza and death. Interestingly, the study detected a short-term harvesting effect between influenza activity and all-cause mortality. Whether such findings can be generalized to other cause-of-death groupings and locations with different circulating influenza strains, health care systems, age structures, and contact patterns needs to be further explored. In addition, previous studies seldom reported potential disparities in influenza-associated excess mortality due to influenza B lineage and sex [19,22].

Guangzhou, a subtropical city in the western Pacific region of southern China (location N 23°8ʹ, E 113°17ʹ), is an international transportation hub with a permanent population of approximately 15 million (in 2018) and an area of 7434.4 km^2^. Free influenza vaccination has been available for older residents since 2021 in Guangzhou; however, the vaccination coverage rate has been unsatisfactory overall in China [23]. Assessment of the mortality burden of influenza would provide necessary data for evaluating this program. The current study aimed to estimate excess mortality associated with influenza virus subtypes and lineages for different cause-of-death groupings in Guangzhou from 2015 to 2018 across different age groups and sexes using DLNMs.

## Methods

### Ethical Considerations

This study was approved by the Research Ethics Committee of Southern Medical University (NFYKDX-ER2022012). The need for informed consent was waived because the data were deidentified and aggregated.

### Data Sources

Individual data for deaths occurring between January 5, 2015, and December 30, 2018, in Guangzhou were obtained from the Guangzhou Center for Disease Control and Prevention (CDC). Individual death information included the underlying cause of death, age at death, and sex. The daily number of deaths was aggregated. Here, we considered 5 cause-of-death groupings associated with influenza virus infection: all causes (International Statistical Classification of Diseases and Related Health Problems, 10th Revision [ICD-10] codes A00-Z99), respiratory and cardiovascular diseases (ICD-10 codes I00-I99 and J00-J99), respiratory disease (ICD-10 codes J00-J99), pneumonia and influenza (ICD-10 codes J10-J18), and cardiovascular disease (ICD-10 codes I00-I99).

The Guangzhou CDC also provided influenza surveillance data, including the weekly proportions of specimens testing positive for influenza A (H1N1), A (H3N2), B/Yamagata, and B/Victoria, and the weekly proportion of consultations for influenza-like illness (ILI), that is, a body temperature ≥38 °C with cough or sore throat, among outpatient visits at sentinel hospitals in Guangzhou.

Data on annual population size were obtained from the Public Security Bureau of Guangzhou Municipality. We collected data on daily mean temperature and relative humidity from the website of the China Meteorological Data Service Center [24].

### Statistical Analysis

We defined a weekly influenza virus activity proxy by multiplying the weekly proportion of consultations for ILI and the proportion of specimens testing positive for different subtypes and lineages, and then multiplying the resulting value by 1000 [25,26]. Then, we converted the weekly proxy to a daily proxy with cubic smoothing splines with 1 *df* per week, determined with generalized cross-validation [21]. The daily population size was estimated by linear interpolation. Daily mean temperature and relative humidity data were used to calculate daily absolute humidity [17], which has been suggested to be a better predictor of influenza virus transmission and survival than relative humidity [27].

A quasi-Poisson regression model was applied to estimate the excess mortality associated with influenza for different cause-of-death groupings, age groups (<60 years, 60-79 years, ≥80 years, and all ages), and sex. The associations of death counts with influenza and temperature were determined using a DLNM [28], as follows:



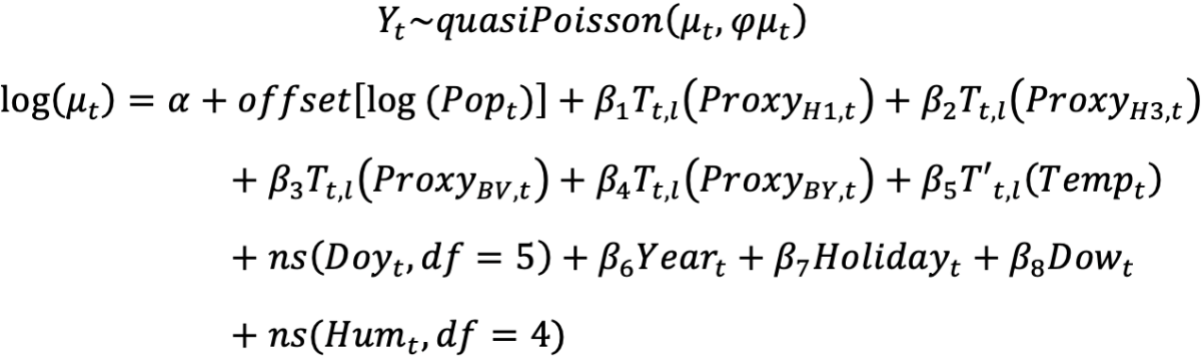



where Y_t_ and μ_t_ were the observed and expected number of deaths on day t, respectively. φ is the overdispersion parameter. The logarithm of population (Pop_t_), with a fixed regression coefficient of 1, was used as an offset. The influenza activity proxy variables for A (H1N1), A (H3N2), B/Victoria, and B/Yamagata on day t were Proxy_H1,t_, Proxy_H3,t_, Proxy_BV,t_, and Proxy_BY,t_, respectively. Cross-basis Tʹ_t,l_(.) was constructed for the activity proxy variable of each influenza subtype/lineage, assuming a linear relationship between influenza virus activity and population mortality [3,6,13,14,21]. Cross-basis Tʹ_t,l_(.) was constructed for daily temperature (Temp_t_) on day t with a natural cubic spline with 4 *df* [29]. It should be noted that the lag-response associations for the temperature and influenza virus activity proxies were set up with natural cubic splines with 3 knots placed at equally spaced log values of lags of 30 days to fully capture possible short-term mortality displacement effects (Multimedia Appendix 1) [21,30].

In addition, we controlled the effect of absolute humidity (Hum_t_) using a natural cubic spline with 4 *df* [22]. Additionally, we included a categorical variable for the day of the week (Dow_t_) and an indicator variable for holidays (Holiday_t_) in the model. Additional adjustments in the model included a natural cubic spline with 5 *df* for the day of the year (Doy_t_; ie, 1-366) and a categorical variable for calendar year (Year_t_), to control for seasonality and the time trend in death counts, respectively [21].

We present the relative risk (RR) and cumulative RR of death associated with an increase of 10 units in the influenza virus activity proxy relative to an influenza activity proxy of 0 across a lag of 0 to 30 days. The daily number of excess deaths related to influenza was estimated as the difference between the estimated number of deaths given the observed influenza virus activity and the estimate given that the influenza virus did not circulate (ie, the influenza virus activity proxy was 0) [22]. The excess mortality rates (EMRs) associated with influenza for different age groups were estimated by dividing the influenza-related excess death numbers by the corresponding population sizes and then multiplying by 100,000. It should be noted that the EMRs associated with influenza in this study were in units per 100,000 persons per year. We estimated the EMRs that incorporated the lagged effects of influenza on mortality over 0 to 30 days. Meanwhile, we applied Monte Carlo simulations to estimate the 95% empirical CI (95% eCI) of the EMRs, which accounted for autocorrelation using the Newey-West method [31-33].

To detect whether there was a sex difference in influenza-related EMRs, we divided the male EMRs by female EMRs to obtain the male-to-female excess mortality ratios (MFRs) and derived the corresponding 95% CIs using the delta method [22].

To check the robustness of the results, we conducted sensitivity analyses by (1) changing the maximum number of lag days for lag-response relationships between the death and activity proxies of each influenza subtype and lineage, as well as for the relationship between death and temperature; (2) changing the way we controlled the temporal trend in the mortality rate; (3) ignoring the classifications of influenza virus types and influenza B lineages; (4) applying different cross-basis matrices to temperature; and (5) including the influenza virus activity proxy and temperature at a lag of 7 days in the model (Multimedia Appendix 1). All analyses were performed in R (version 4.1.1; R Foundation for Statistical Computing).

## Results

**Figure 1:**
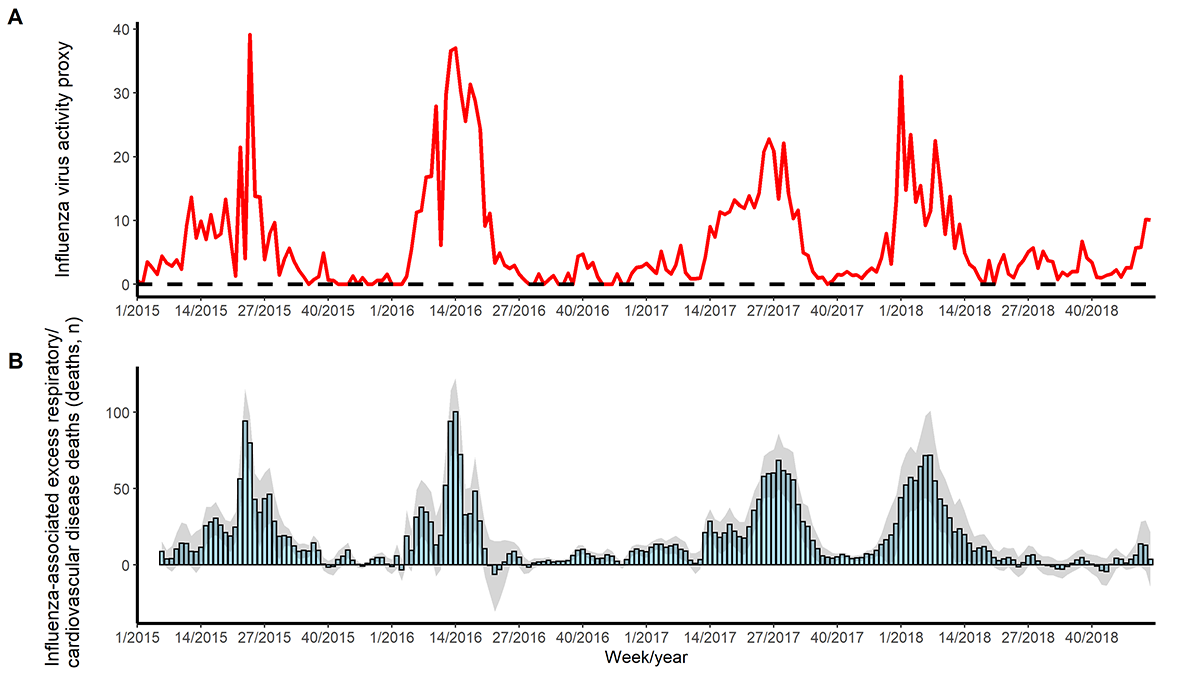
Influenza activity and weekly influenza-associated excess respiratory and cardiovascular deaths in Guangzhou, China, from 2015 to 2018. (A) Influenza virus activity proxy (this proxy does not have units). (B) Weekly influenza-associated excess respiratory/cardiovascular deaths. The bars in B represent the estimates of excess influenza-related deaths per week and the shaded areas are the corresponding 95% empirical CIs. The influenza virus activity proxy was calculated by multiplying the weekly proportion of consultations for influenza-like illness and the proportion of specimens testing positive for different subtypes and lineages, and then multiplying the resulting value by 1000.

There were 199,777 death certificates (86,440 for women and 113,337 for men; 34,229 for the 0-to-59-year age group, 73,552 for the 60-to-79-year age group, and 91,996 for the ≥80-year age group) included in this analysis, of which 105,998 were respiratory/cardiovascular disease deaths, 28,528 were respiratory disease deaths, and 12,255 were pneumonia/influenza deaths. In Guangzhou from 2015 to 2018, influenza viruses circulated every year. In 2015 and 2017, influenza activity peaked in the summer, while in 2016 and 2018, influenza activity was greater in the winter and early spring. A similar pattern was observed for the excess respiratory/cardiovascular disease mortality associated with influenza (Figure 1).

It was estimated that 2198 all-cause deaths, 1786 respiratory/cardiovascular disease deaths, 825 respiratory disease deaths, and 340 pneumonia/influenza deaths were attributable to influenza annually; correspondingly, the annual influenza-associated excess all-cause, respiratory/cardiovascular disease, respiratory disease, and pneumonia/influenza mortality rates were 25.06 (95% eCI 19.85-30.16), 20.36 (95% eCI 16.75-23.74), 9.41 (95% eCI 7.78-10.90) and 3.88 (95% eCI 2.80-4.84) per 100,000 persons, respectively (Table 1). Excess deaths associated with influenza accounted for 4.4% (8791/199,777), 6.7% (7142/105,998), 11.6% (3301/28,528), and 11.1% (1361/12,255) of all-cause, respiratory/cardiovascular disease, respiratory disease, and pneumonia/influenza deaths, respectively. Influenza-associated excess deaths due to respiratory/cardiovascular disease, respiratory disease, and pneumonia/influenza accounted for 81.2% (7142/8791), 37.6% (3301/8791), and 15.5% (1361/8791) of excess all-cause deaths, respectively.

The excess mortality due to various underlying causes associated with influenza varied across age groups. The EMR for each cause of death associated with influenza in people aged ≥80 years was higher than that in people aged 60 to 79 years. However, it should be noted that only influenza-related excess mortality due to respiratory/cardiovascular disease was statistically significant among deaths from all different causes in the 0-to-59-year age group. The EMRs for respiratory/cardiovascular disease for people aged 60 to 79 years and ≥80 years were 45.21 (95% eCI 33.31-56.74) and 452.63 (95% eCI 357.46-544.47) per 100,000 persons, respectively, which was higher than the EMR for people aged 0 to 59 years (91.50, 95% eCI 4.79-165.41). The burden in these two age groups represented 32.9% (2346/7142) and 63.7% (4549/7142) of the excess respiratory/cardiovascular disease deaths attributed to influenza, respectively (Table 1).

**Table 1:** Average annual excess mortality rates associated with influenza by cause-of-death grouping and age group in Guangzhou, China, from 2015 to 2018.

Age groups (years) and cause of death		Deaths, n (95% eCI^a^)	Rate per 100,000 persons (95% eCI)
**0-59**			
	All causes	64.05 (–105.30 to 226.41)	0.89 (–1.46 to 3.14)
	Respiratory/cardiovascular disease	91.50 (4.79 to 165.41)	1.27 (0.07 to 2.29)
	Respiratory disease	32.42 (–0.99 to 56.29)	0.45 (–0.01 to 0.78)
	Pneumonia/influenza	16.80 (–13.76 to 35.73)	0.23 (–0.19 to 0.49)
	Cardiovascular disease	55.53 (–23.09 to 122.85)	0.77 (–0.32 to 1.70)
**60-79**			
	All causes	856.96 (621.47 to 1086.86)	66.06 (47.91 to 83.79)
	Respiratory/cardiovascular disease	586.48 (432.06 to 735.96)	45.21 (33.31 to 56.74)
	Respiratory disease	290.12 (223.20 to 344.15)	22.37 (17.21 to 26.53)
	Pneumonia/influenza	103.51 (60.04 to 136.67)	7.98 (4.63 to 10.54)
	Cardiovascular disease	283.58 (144.93 to 416.85)	21.86 (11.17 to 32.13)
≥**80**			
	All causes	1305.53 (996.43 to 1596.4)	519.56 (396.54 to 635.31)
	Respiratory/cardiovascular disease	1137.35 (898.22 to 1368.13)	452.63 (357.46 to 544.47)
	Respiratory disease	509.81 (384.01 to 626)	202.89 (152.82 to 249.13)
	Pneumonia/influenza	222.58 (140.17 to 293.36)	88.58 (55.78 to 116.75)
	Cardiovascular disease	619.02 (433.25 to 796.09)	246.35 (172.42 to 316.82)
**All**			
	All causes	2197.87 (1740.75 to 2644.78)	25.06 (19.85 to 30.16)
	Respiratory/cardiovascular disease	1785.47 (1469.03 to 2082.24)	20.36 (16.75 to 23.74)
	Respiratory disease	825.26 (682.51 to 955.75)	9.41 (7.78 to 10.90)
	Pneumonia/influenza	340.34 (245.92 to 424.18)	3.88 (2.80 to 4.84)
	Cardiovascular disease	940.06 (696.35 to 1171.28)	10.72 (7.94 to 13.36)

^a^eCI: empirical CI.

The difference in influenza-associated excess respiratory/cardiovascular disease mortality rate by sex was not statistically significant. However, the excess respiratory mortality was significantly higher in males than in females, with an MFR of 1.34 (95% CI 1.17-1.54). Conversely, the MFR of influenza-associated EMR for cardiovascular disease was 0.72 (95% CI 0.63-0.82; Table 2). Furthermore, the MFR varied by age group. For excess respiratory deaths related to influenza among those aged 60 to 79 and ≥80 years, the MFRs were 3.03 (95% CI 2.75-3.33) and 1.45 (95% CI 1.41-1.49), respectively, and the MFR was 0.45 (95% CI 0.23-0.89) for people aged 0 to 59 years (Multimedia Appendix 2). For people aged 0 to 59 and 60 to 79 years, the MFRs for excess cardiovascular deaths were 3.24 (95% CI 1.96-5.34) and 2.05 (95% CI 1.90-2.22), respectively. However, for influenza-associated excess cardiovascular deaths in people aged ≥80 years, the MFR was 0.65 (95% CI 0.64-0.67) (Multimedia Appendix 3).

**Figure 2:**
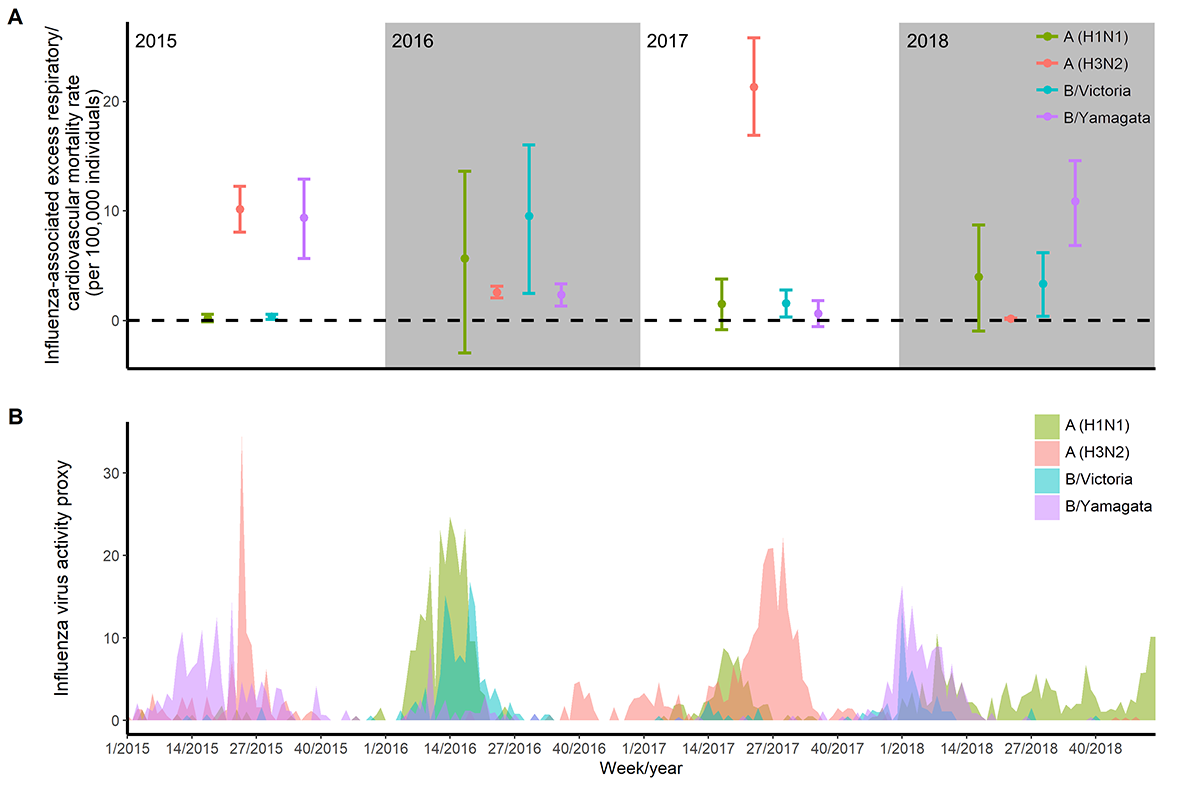
Annual influenza-associated excess respiratory/cardiovascular mortality rates and influenza activity in Guangzhou, China, from 2015 to 2018. (A) Annual influenza-associated excess respiratory/cardiovascular mortality rates per 100,000 persons by influenza subtype/lineage. (B) Influenza virus activity proxies by influenza subtype/lineage (this proxy does not have units). The dots in A indicate point estimates of excess respiratory/cardiovascular mortality, while vertical line segments indicate the corresponding 95% empirical CIs.

Temporal trends of influenza virus activity varied by subtype and lineage (Figure 2). The EMR for respiratory/cardiovascular disease attributed to influenza A (H3N2) was 8.47 (95% eCI 6.60-10.30) per 100,000 persons, which was higher than the EMR due to influenza A (H1N1) (2.83, 95% eCI –1.26 to 6.71), B/Victoria (3.68, 95% eCI 0.81-6.49), and B/Yamagata (5.81, 95% eCI 3.35-8.25). For people aged 60 to 79 years and those aged ≥80 years, the excess respiratory/cardiovascular disease mortality associated with A (H3N2) was higher than that for other influenza subtypes or lineages. However, for people aged 0 to 59 years, excess influenza-related deaths were dominated by influenza A (H1N1), with excess mortality of 1.29 (95% eCI 0.07-2.32; Table 3).

Between 2015 and 2018, the predominant circulating influenza virus subtypes and lineages varied. Correspondingly, the EMRs per 100,000 persons for respiratory and cardiovascular disease associated with influenza A (H1N1), A (H3N2), B/Victoria, and B/Yamagata ranged between 0.21 (95% eCI –0.13 to 0.54) and 5.62 (95% eCI –2.74 to 13.19), between 0.15 (95% eCI 0.10-0.19) and 21.25 (95% eCI 16.61-25.59), between 0.32 (95% eCI 0.09-0.56) and 9.52 (95% eCI 2.58-16.29), and between 0.63 (95% eCI –0.62 to 1.77) and 10.84 (95% eCI 6.74-14.83), respectively (Figure 2).

**Figure 3:**
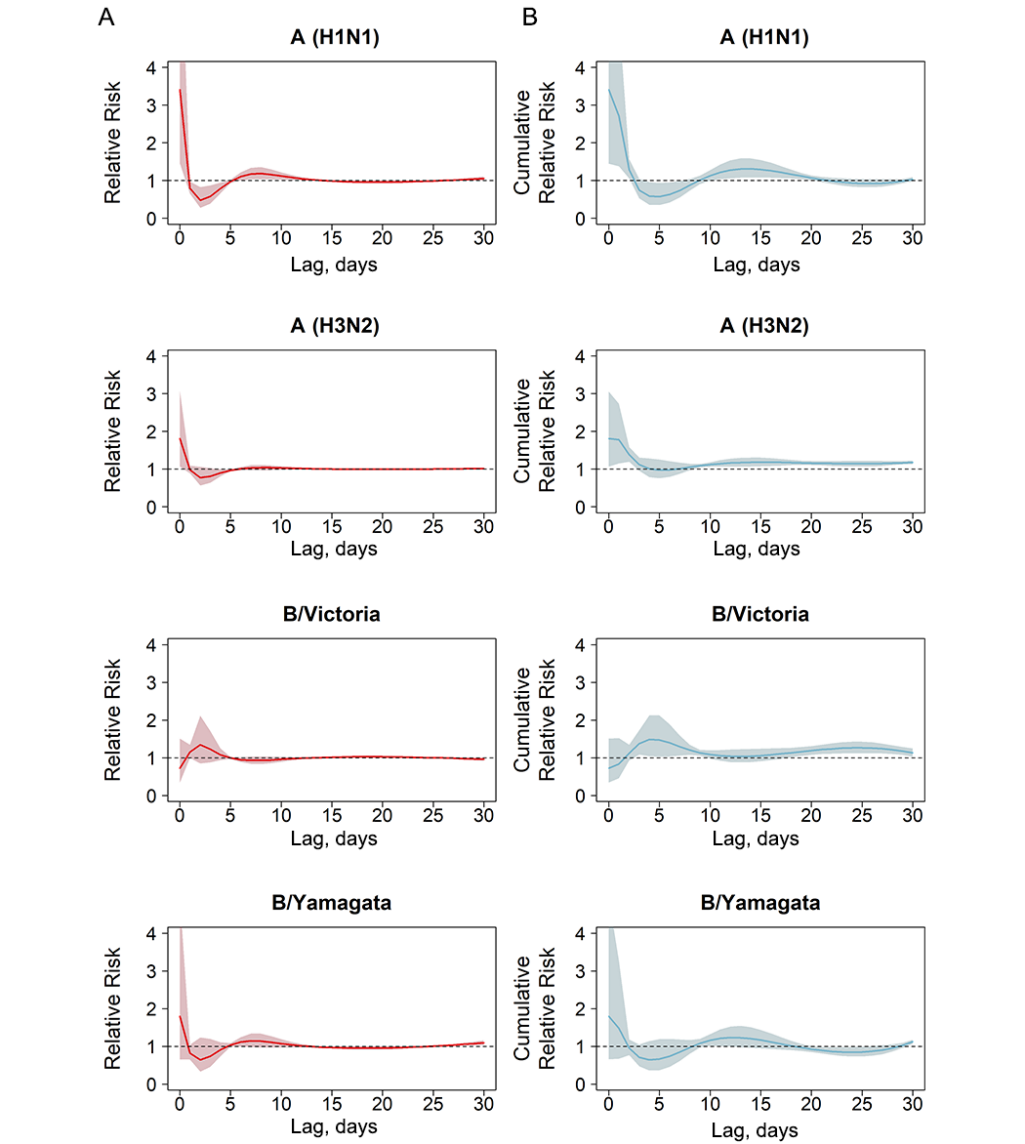
Relative risks and cumulative relative risks of death associated with influenza subtypes/lineages. (A) Relative risks of death on each single day and (B) cumulative relative risks of death associated with an increase of 10 units in influenza virus activity proxy for influenza A (H1N1), A (H3N2), B/Victoria, and B/Yamagata. The solid lines represent the estimates of relative risk over time, and the shaded areas are the corresponding 95% empirical CIs.

The RR of respiratory/cardiovascular disease death associated with influenza virus subtypes/lineages changed with lag time. For influenza A (H1N1), the highest RR occurred on the current day, and there seemed to be a displacement effect during days 2 to 5. This effect lasted approximately 14 days. Similar patterns were observed in the RRs for influenza A (H3N2) and B/Yamagata. For B/Victoria, RR peaked at a lag of 3 days. The displacement effect was observed on days 5 to 13 (Figure 3).

**Table 2:** Average annual excess mortality associated with influenza by cause-of-death grouping and sex in Guangzhou, China, from 2015-2018.

Underlying cause	Male		Female		Male-to-female excess mortality ratio (95% CI)	*P* value
	Deaths, n (95% eCI^a^)	Rate per 100,000 persons (95% eCI)	Deaths, n (95% eCI)	Rate per 100,000 persons (95% eCI)		
All causes	1060.79 (755.56-1358.70)	24.12 (17.18-30.89)	1132.64 (859.06-1385.33)	25.91 (19.65-31.69)	0.93 (0.86-1.01)	.09
Respiratory/ cardiovascular diseases	876.90 (674.64-1071.51)	19.94 (15.34-24.36)	906.37 (716.96-1086.57)	20.73 (16.40-24.85)	0.96 (0.88-1.06)	.41
Respiratory diseases	472.95 (362.98-571.77)	10.75 (8.25-13.00)	351.06 (263.27-428.41)	8.03 (6.02-9.80)	1.34 (1.17-1.54)	<.001
Pneumonia/ influenza	177.27 (109.82-232.72)	4.03 (2.50-5.29)	162.02 (100.26-216.15)	3.71 (2.29-4.94)	1.09 (0.88-1.34)	.46
Cardiovascular diseases	393.08 (227.15-553.88)	8.94 (5.16-12.59)	543.79 (375.64-705.39)	12.44 (8.59-16.14)	0.72 (0.63-0.82)	<.001

^a^eCI: empirical CI.

**Table 3:** Excess respiratory and cardiovascular mortality rates associated with different influenza subtypes and lineages.

Influenza type or lineage and age groups (years)		Rate per 100,000 persons (95% eCI^a^)
**A (H1N1)**		
	0-59	1.29 (0.07 to 2.32)
	60-79	5.74 (–8.61 to 19.15)
	≥80	55.75 (–60.87 to 166.10)
	All ages	2.83 (–1.26 to 6.71)
**A (H3N2)**		
	0-59	0.33 (–0.24 to 0.85)
	60-79	20.22 (14.56 to 25.63)
	≥80	180.15 (130.75 to 227.38)
	All ages	8.47 (6.60 to 10.30)
**B/Victoria**		
	0-59	–0.47 (–1.59 to 0.45)
	60-79	7.36 (–2.87 to 16.65)
	≥80	88.82 (6.18 to 168.44)
	All ages	3.68 (0.81 to 6.49)
**B/Yamagata**		
	0-59	0.02 (–0.78 to 0.74)
	60-79	12.87 (4.60 to 20.74)
	≥80	138.21 (70.53 to 204.40)
	All ages	5.81 (3.35 to 8.25)
**All influenza**		
	0-59	1.27 (0.07 to 2.29)
	60-79	45.21 (33.31 to 56.74)
	≥80	452.63 (357.46 to 544.47)
	All ages	20.36 (16.75 to 23.74)

^a^eCI: empirical CI.

Estimates of influenza-associated excess respiratory and cardiovascular disease mortality rates did not change significantly after changing the method of controlling for the temporal trend in mortality rate, changing the maximum lag, or the function used for the lag-response dimension in the cross-basis matrices of influenza and temperature, ignoring the classification of influenza B lineages (Multimedia Appendix 4).

## Discussion

### Principal Results

In this study, we report excess mortality associated with influenza and its subtypes/lineages from 2015 to 2018 in Guangzhou, a subtropical city of China. We estimate that 1786 respiratory/cardiovascular disease deaths were attributable to influenza annually, accounting for 6.7% (7142/105,998) of all respiratory/cardiovascular disease deaths. We found that influenza-associated excess mortality was higher in the elderly, which is consistent with the conclusions of other studies [1,14,15,21,34].

Variations were observed in the estimates of influenza-associated excess mortality by subtype/lineage. The mortality burden of influenza B/Yamagata was higher than that of B/Victoria, which is consistent with previous studies [6,22,34]. The disparity between the lineages may be due to the elderly being more likely to be infected by influenza B/Yamagata than B/Victoria [35,36]. In 2018, influenza B/Yamagata was the predominant lineage in Guangzhou, and the vast majority of influenza-related excess deaths were attributed to B/Yamagata. Similarly, an outbreak dominated by influenza B/Yamagata was reported in Europe during the 2017-2018 influenza season, causing substantial excess mortality [34]. Together, the evidence implies that influenza B does not necessarily lead to mild illness in humans; the mortality burden of influenza B cannot be ignored. Furthermore, including influenza B lineages in active surveillance would help better understand the epidemiology of influenza B and reduce the excess mortality of influenza by enabling targeted interventions. Vaccination is the most effective way to prevent influenza; however, the vaccination coverage in China is low (9.4%) [23], partly because influenza vaccination is not government funded in many places [37]. During the 2018-2019 influenza season, the quadrivalent inactivated vaccine was approved for the first time in China [38]. In 2019, 30.78 million doses of influenza vaccines were distributed in China, but most of them were trivalent inactivated vaccines [37]. Compared with the trivalent influenza vaccine, the quadrivalent influenza vaccine has a higher antibody protection rate and antibody-positive seroconversion rate against the increased influenza B strain [39,40]. High-risk groups are recommended to receive quadrivalent influenza vaccines when vaccines are available, given the nonnegligible burden of influenza B.

We also noted that the mortality burden of influenza virus subtypes and lineages differed according to age group. For people aged ≥60 years, the fatality rate caused by influenza A (H3N2) was higher than that of A (H1N1), B/Victoria, and B/Yamagata. The risk of influenza virus infection and severe complications, including death, is increased by the stronger antigenic drift and virulence of influenza A (H3N2) [22,41]. However, for people aged 0 to 59 years, influenza A (H1N1) had a greater EMR than other influenza subtypes or lineages, which is in accord with previous studies [22]. The lower mortality burden of influenza A (H1N1) might be due to protection from early life exposure to this subtype [42].

Consistently with previous studies performed in Shanghai, we did not find a statistically significant difference in influenza-related excess respiratory/cardiovascular disease deaths between males and females [22]. However, males had a higher influenza-related excess respiratory mortality rate than females and a lower excess cardiovascular mortality rate. In addition, in people aged 60 to 79 years, it has been found that the influenza-related EMR for respiratory disease or cardiovascular disease in men is higher than in women, and the same situation was found in people aged 65 to 74 years in a US study [19]. However, for people aged ≥80 years, men had higher influenza-related excess respiratory mortality than women, but a lower excess cardiovascular mortality associated with influenza. In the US study, the results of a comparison of point estimates of EMR for the corresponding causes for males and females were consistent with our results, but the difference was not statistically significant [19]. Decreasing testosterone levels and higher respiratory mortality in older men [22,36], or the fact that women may have a different innate or acquired immune response than men due to discrepancies in sex steroid hormones [43], might lead to sex disparities in the severity of illness after influenza virus infection.

Our estimate of average annual influenza-associated excess all-cause mortality (25.06 per 100,000 individuals) was higher than that of previous studies in countries/cities and time periods including Greece from 2013 to 2017 (23.60) [21], Beijing from 2007 to 2013 (19.10) [14], Guangzhou from 2010 to 2012 (14.72) [15], and the United States from 1997 to 2007 (11.78) [19]. However, the estimate was relatively lower than the number of annual average influenza-related excess all-cause deaths in Europe for 2017-2018 (25.40) [34], Chongqing from 2012 to 2018 (33.50) [44], and Shanghai from 2010 to 2015 (27.66) [22]. Factors such as population structure, health status, health care capacity, circulating strains, host immunity, public health interventions (eg, influenza vaccination programs), and social distancing might contribute to these differences [20,22]. In addition to the abovementioned factors that may lead to heterogeneity in the results of different studies, some methodological differences need to be considered. The estimates of influenza-associated excess mortality varied with different analytical strategies, although the disparities were not significant in the sensitivity analysis. Influenza can be divided into different subtypes and lineages to explore the impact of influenza on the health of populations when relevant data are available, which also helps us understand the effects of different influenza subtypes and lineages on mortality.

Previous studies have estimated excess mortality based on the association between influenza activity proxies at a lag of 0, 1, or 2 weeks and mortality [20]. In this study, we used a DLNM to capture the association between influenza and mortality. A sensitivity analysis showed no significant differences between the influenza-associated excess respiratory/cardiovascular disease mortality rates estimated by a DLNM and a model that included linear terms of an influenza virus activity proxy and a natural cubic spline of temperature at a lag of 7 days. DLNMs allow us to consider a flexible lag-response relationship between influenza and death. The time course of influenza effects and potential mortality displacement can be examined with DLNMs [21,30]. We found that many influenza-related respiratory/cardiovascular disease deaths occurred during the initial exposure to influenza A (H1N1), A (H3N2), and B/Yamagata. Therefore, starting antiviral treatment as soon as possible, especially with neuraminidase inhibitors and other means of treatment for critically ill influenza patients, is likely to bring great survival benefits [35]. Taking into account the observed mortality displacement, we estimate that 743, 322, and 510 annual respiratory/cardiovascular disease deaths in Guangzhou were attributable to influenza A (H3N2), B/Victoria, and B/Yamagata, respectively; this indicates that the impact of influenza A (H3N2) and B on mortality is not limited to bringing forward deaths in the short term. Therefore, the mortality burden of influenza should not be overlooked. Efforts are needed to raise awareness of influenza as a severe disease, especially among high-risk groups.

### Limitations

This study had some limitations. First, we did not use age-specific influenza virus activity proxies to estimate influenza-associated excess mortality, and a proxy for all-age activity was used instead. Second, the study period was relatively short (ie, 4 years), which may have influenced the comparison of the mortality burden of different influenza subtypes and lineages. This is mainly because the virology data included samples that tested positive for influenza B, but information on lineage was unavailable before 2015. Data from 2019 onwards were not collected. Moreover, we did not consider the influence of factors such as respiratory syncytial virus and vaccination coverage on the estimate of influenza-associated excess mortality, since such data were not available.

### Conclusion

In conclusion, after considering the observed mortality displacement, influenza was associated with substantial mortality in Guangzhou, occurring predominantly in the elderly. The mortality burden of influenza B, particularly B/Yamagata, cannot be ignored. Contrasting sex differences were found in influenza-associated excess mortality from respiratory diseases and from cardiovascular diseases, and the underlying mechanisms need to be investigated in further studies. Our findings can help us better understand the magnitude and time-course of the effect of influenza on mortality and inform targeted interventions for mitigating the influenza mortality burden, such as immunizations with quadrivalent vaccines (especially for older individuals), behavioral campaigns, and treatment strategies.

## Appendix

Multimedia Appendix 1 Additional details of the methods.

Multimedia Appendix 2 Average annual excess respiratory mortality rates related to influenza by sex and age.

Multimedia Appendix 3 Average annual excess cardiovascular mortality rates related to influenza by sex and age.

Multimedia Appendix 4 Comparison of average annual excess respiratory and cardiovascular mortality rates attributable to all influenza estimated from different models in the sensitivity analysis.

## Abbreviations

CDC: Center for Disease Control and Prevention
DLNM: distributed-lag nonlinear model
EMR: excess mortality rate
ILI: influenza-like illness
MFR: male-to-female excess mortality ratio
RR: relative risk

## Data Availability

The data sets generated during and/or analyzed during the current study are available from the corresponding author on reasonable request.

## References

[1] Iuliano AD, Roguski KM, Chang HH, Muscatello DJ, Palekar R, Tempia S, Cohen C, Gran JM, Schanzer D, Cowling BJ, Wu P, Kyncl J, Ang LW, Park M, Redlberger-Fritz M, Yu H, Espenhain L, Krishnan A, Emukule G, van Asten L, Pereira da Silva S, Aungkulanon S, Buchholz U, Widdowson M, Bresee JS, Global Seasonal Influenza-associated Mortality Collaborator Network (2018). Estimates of global seasonal influenza-associated respiratory mortality: a modelling study. Lancet.

[2] Influenza (Seasonal). World Health Organization.

[3] Li L, Liu Y, Wu P, Peng Z, Wang X, Chen T, Wong JYT, Yang J, Bond HS, Wang L, Lau YC, Zheng J, Feng S, Qin Y, Fang VJ, Jiang H, Lau EHY, Liu S, Qi J, Zhang J, Yang J, He Y, Zhou M, Cowling BJ, Feng L, Yu H (2019). Influenza-associated excess respiratory mortality in China, 2010-15: a population-based study. Lancet Public Health.

[4] Grohskopf LA, Alyanak E, Ferdinands JM, Broder KR, Blanton LH, Talbot HK, Fry AM (2021). Prevention and control of seasonal influenza with vaccines: recommendations of the Advisory Committee on Immunization Practices, United States, 2021-22 influenza season. MMWR Recomm Rep.

[5] Coleman BL, Fadel SA, Fitzpatrick T, Thomas S (2018). Risk factors for serious outcomes associated with influenza illness in high- versus low- and middle-income countries: Systematic literature review and meta-analysis. Influenza Other Respir Viruses.

[6] Yu X, Wang C, Chen T, Zhang W, Yu H, Shu Y, Hu W, Wang X (2017). Excess pneumonia and influenza mortality attributable to seasonal influenza in subtropical Shanghai, China. BMC Infect Dis.

[7] Feng L, Shay DK, Jiang Y, Zhou H, Chen X, Zheng Y, Jiang L, Zhang Q, Lin H, Wang S, Ying Y, Xu Y, Wang N, Feng Z, Viboud C, Yang W, Yu H (2012). Influenza-associated mortality in temperate and subtropical Chinese cities, 2003-2008. Bull World Health Organ.

[8] Liu X, Qin G, Li X, Zhang J, Zhao K, Hu M, Wang X (2017). Excess mortality associated with influenza after the 2009 H1N1 pandemic in a subtropical city in China, 2010-2015. Int J Infect Dis.

[9] Yu H, Feng L, Viboud CG, Shay DK, Jiang Y, Zhou H, Zhou M, Xu Z, Hu N, Yang W, Nie S (2013). Regional variation in mortality impact of the 2009 A(H1N1) influenza pandemic in China. Influenza Other Respir Viruses.

[10] Hardelid P, Pebody R, Andrews N (2013). Mortality caused by influenza and respiratory syncytial virus by age group in England and Wales 1999-2010. Influenza Other Respir Viruses.

[11] Green HK, Andrews N, Fleming D, Zambon M, Pebody R (2013). Mortality attributable to influenza in England and Wales prior to, during and after the 2009 pandemic. PLoS One.

[12] van Asten L, van den Wijngaard C, van Pelt W, van de Kassteele J, Meijer A, van der Hoek W, Kretzschmar M, Koopmans M (2012). Mortality attributable to 9 common infections: significant effect of influenza A, respiratory syncytial virus, influenza B, norovirus, and parainfluenza in elderly persons. J Infect Dis.

[13] Thompson WW, Shay DK, Weintraub E, Brammer L, Cox N, Anderson LJ, Fukuda K (2003). Mortality associated with influenza and respiratory syncytial virus in the United States. JAMA.

[14] Wu S, Wei Z, Greene CM, Yang P, Su J, Song Y, Iuliano AD, Wang Q (2018). Mortality burden from seasonal influenza and 2009 H1N1 pandemic influenza in Beijing, China, 2007-2013. Influenza Other Respir Viruses.

[15] Wang H, Fu C, Li K, Lu J, Chen Y, Lu E, Xiao X, Di B, Liu H, Yang Z, Wang M (2014). Influenza associated mortality in Southern China, 2010-2012. Vaccine.

[16] Guo R, Zheng H, Ou C, Huang L, Zhou Y, Zhang X, Liang C, Lin J, Zhong H, Song T, Luo H (2016). Impact of influenza on outpatient visits, hospitalizations, and deaths by using a time series Poisson generalized additive model. PLoS One.

[17] Wu P, Goldstein E, Ho LM, Yang L, Nishiura H, Wu JT, Ip DKM, Chuang S, Tsang T, Cowling BJ (2012). Excess mortality associated with influenza A and B virus in Hong Kong, 1998-2009. J Infect Dis.

[18] Thompson WW, Weintraub E, Dhankhar P, Cheng P, Brammer L, Meltzer MI, Bresee JS, Shay DK (2009). Estimates of US influenza-associated deaths made using four different methods. Influenza Other Respir Viruses.

[19] Quandelacy TM, Viboud C, Charu V, Lipsitch M, Goldstein E (2014). Age- and sex-related risk factors for influenza-associated mortality in the United States between 1997-2007. Am J Epidemiol.

[20] Li L, Wong JY, Wu P, Bond HS, Lau EHY, Sullivan SG, Cowling BJ (2018). Heterogeneity in estimates of the impact of influenza on population mortality: a systematic review. Am J Epidemiol.

[21] Lytras T, Pantavou K, Mouratidou E, Tsiodras S (2019). Mortality attributable to seasonal influenza in Greece, 2013 to 2017: variation by type/subtype and age, and a possible harvesting effect. Euro Surveill.

[22] Jin S, Li J, Cai R, Wang X, Gu Z, Yu H, Fang B, Chen L, Wang C (2020). Age- and sex-specific excess mortality associated with influenza in Shanghai, China, 2010-2015. Int J Infect Dis.

[23] Wang Q, Yue N, Zheng M, Wang D, Duan C, Yu X, Zhang X, Bao C, Jin H (2018). Influenza vaccination coverage of population and the factors influencing influenza vaccination in mainland China: A meta-analysis. Vaccine.

[24] China Meteorological Data Service Centre. National Meteorological Information Centre.

[25] Goldstein E, Cobey S, Takahashi S, Miller JC, Lipsitch M (2011). Predicting the epidemic sizes of influenza A/H1N1, A/H3N2, and B: a statistical method. PLoS Med.

[26] Goldstein E, Viboud C, Charu V, Lipsitch M (2012). Improving the estimation of influenza-related mortality over a seasonal baseline. Epidemiology.

[27] Shaman J, Kohn M (2009). Absolute humidity modulates influenza survival, transmission, and seasonality. Proc Natl Acad Sci U S A.

[28] Gasparrini A, Armstrong B, Kenward MG (2010). Distributed lag non-linear models. Stat Med.

[29] Guo Y, Gasparrini A, Armstrong BG, Tawatsupa B, Tobias A, Lavigne E, Coelho MDSZS, Pan X, Kim H, Hashizume M, Honda Y, Guo YL, Wu C, Zanobetti A, Schwartz JD, Bell ML, Overcenco A, Punnasiri K, Li S, Tian L, Saldiva P, Williams G, Tong S (2016). Temperature variability and mortality: a multi-country study. Environ Health Perspect.

[30] Zanobetti A, Wand MP, Schwartz J, Ryan LM (2000). Generalized additive distributed lag models: quantifying mortality displacement. Biostatistics.

[31] Gasparrini A, Leone M (2014). Attributable risk from distributed lag models. BMC Med Res Methodol.

[32] Bottomley C, Scott J, Isham V (2019). Analysing interrupted time series with a control. Epidemiol Methods.

[33] Newey WK, West KD (1987). A simple, positive semi-definite, heteroskedasticity and autocorrelation consistent covariance matrix. Econometrica.

[34] Nielsen J, Vestergaard LS, Richter L, Schmid D, Bustos N, Asikainen T, Trebbien R, Denissov G, Innos K, Virtanen MJ, Fouillet A, Lytras T, Gkolfinopoulou K, Heiden MAD, Grabenhenrich L, Uphoff H, Paldy A, Bobvos J, Domegan L, O'Donnell J, Scortichini M, de Martino A, Mossong J, England K, Melillo J, van Asten L, de Lange MM, Tønnessen R, White RA, da Silva SP, Rodrigues AP, Larrauri A, Mazagatos C, Farah A, Carnahan AD, Junker C, Sinnathamby M, Pebody RG, Andrews N, Reynolds A, McMenamin J, Brown CS, Adlhoch C, Penttinen P, Mølbak K, Krause TG (2019). European all-cause excess and influenza-attributable mortality in the 2017/18 season: should the burden of influenza B be reconsidered?. Clin Microbiol Infect.

[35] Caini S, Kusznierz G, Garate VV, Wangchuk S, Thapa B, de Paula Júnior Francisco José, Ferreira de Almeida WA, Njouom R, Fasce RA, Bustos P, Feng L, Peng Z, Araya JL, Bruno A, de Mora D, Barahona de Gámez Mónica Jeannette, Pebody R, Zambon M, Higueros R, Rivera R, Kosasih H, Castrucci MR, Bella A, Kadjo HA, Daouda C, Makusheva A, Bessonova O, Chaves SS, Emukule GO, Heraud J, Razanajatovo NH, Barakat A, El Falaki F, Meijer A, Donker GA, Huang QS, Wood T, Balmaseda A, Palekar R, Arévalo Brechla Moreno, Rodrigues AP, Guiomar R, Lee VJM, Ang LW, Cohen C, Treurnicht F, Mironenko A, Holubka O, Bresee J, Brammer L, Le MTQ, Hoang PVM, El Guerche-Séblain Clotilde, Paget J, Global Influenza B Study team (2019). The epidemiological signature of influenza B virus and its B/Victoria and B/Yamagata lineages in the 21st century. PLoS One.

[36] Skowronski DM, Chambers C, De Serres G, Sabaiduc S, Winter A, Dickinson JA, Gubbay JB, Fonseca K, Drews SJ, Charest H, Martineau C, Krajden M, Petric M, Bastien N, Li Y (2017). Age-related differences in influenza b infection by lineage in a community-based sentinel system, 2010-2011 to 2015-2016, Canada. J Infect Dis.

[37] Tao Y, Li J, Hu Y, Hu Y, Zeng G, Zhu F (2021). Quadrivalent influenza vaccine (Sinovac Biotech) for seasonal influenza prophylaxis. Expert Rev Vaccines.

[38] Yang XK, Zhang YQ, Feng D, Xia Z, Fan SM, Zhao HT (2021). Characteristics of influenza vaccines lot release in China, 2007 to 2020. Int J Virol.

[39] Moa AM, Chughtai AA, Muscatello DJ, Turner RM, MacIntyre CR (2016). Immunogenicity and safety of inactivated quadrivalent influenza vaccine in adults: A systematic review and meta-analysis of randomised controlled trials. Vaccine.

[40] Bekkat-Berkani R, Ray R, Jain VK, Chandrasekaran V, Innis BL (2016). Evidence update: GlaxoSmithKline's inactivated quadrivalent influenza vaccines. Expert Rev Vaccines.

[41] Rambaut A, Pybus OG, Nelson MI, Viboud C, Taubenberger JK, Holmes EC (2008). The genomic and epidemiological dynamics of human influenza A virus. Nature.

[42] Gostic KM, Bridge R, Brady S, Viboud C, Worobey M, Lloyd-Smith JO (2019). Childhood immune imprinting to influenza A shapes birth year-specific risk during seasonal H1N1 and H3N2 epidemics. PLoS Pathog.

[43] Furman D, Hejblum BP, Simon N, Jojic V, Dekker CL, Thiébaut Rodolphe, Tibshirani RJ, Davis MM (2014). Systems analysis of sex differences reveals an immunosuppressive role for testosterone in the response to influenza vaccination. Proc Natl Acad Sci USA.

[44] Qi L, Li Q, Ding X, Gao Y, Ling H, Liu T, Xiong Y, Su K, Tang W, Feng L, Liu Q (2020). Mortality burden from seasonal influenza in Chongqing, China, 2012-2018. Hum Vaccin Immunother.

