# Supplementary material for: Influenza-Associated Excess Mortality by Age, Sex, and Subtype/Lineage: Population-Based Time-Series Study With a Distributed-Lag Nonlinear Model
Source: JMIR Public Health Surveill. 2023 Jan 11;9:e42530. doi: 10.2196/42530 (PMC9878364; doi:10.2196/42530)
Supplement: Multimedia Appendix 1 [file publichealth_v9i1e42530_app1.pdf]

Supplement Content

Influenza-associated excess mortality by age, sex, and subtype/lineage in a subtropical city in China, 2015-2018: a population-based time-series study with a distributed lag non-linear model

eMethod 1 Descriptions of influenza surveillance data

eMethod 2 Excess mortality rate and its empirical confidence interval

eMethod 3 Confidence interval of male to female rates ratios

eMethod 4 Sensitivity analysis

eMethod 5 Mortality displacement

eFigure 1 Partial autocorrelation function for the residuals of models fitted for death from different reasons in population of different age groups or sex

eMethod 1 Descriptions of influenza surveillance data

Influenza-like illness surveillance: For the surveillance of influenza-like illness (with temperature ≥38°C, accompanied by cough or sore throat), the surveillance points were hospitals or outpatient departments (19 hospitals and outpatient departments were selected from the whole city). Among all the hospital surveillance points, the Children's Hospital of Guangzhou Women and Children's Medical Center carried out surveillance in all consultation rooms of pediatric outpatient clinics, pediatric emergency clinics, fever clinics, and other surveillance sites of hospitals or outpatient departments for influenza-like illness in the internal medicine outpatient service, internal medicine emergency service, fever outpatient service, pediatric outpatient service, and pediatric emergency service. Medical outpatient surveillance should include all offices of the department of internal medicine and infectious diseases.

According to the definition of influenza-like illness, the medical staff in the consulting room of surveillance hospitals or outpatient departments should register the number of influenza-like cases and the total number of outpatient and emergency cases every day by the department.

Virologic surveillance: In addition to the surveillance of influenza-like illnesses, four national surveillance point hospitals (the First People's Hospital of Guangzhou, Children's Hospital of Guangzhou Women and Children Medical Center, Guangzhou Red Cross Hospital, and Liwan Hospital of the Third Affiliated Hospital of Guangzhou Medical University) also carried out virologic surveillance using pharyngeal swabs and respiratory tract swabs or bronchial lavage fluid to collect samples. Hospitals are required to carry out virologic surveillance to collect at least 20 outpatient samples per week and at least 1040 samples per year. Madin-darby canine kidney (MDCK) cells and/or chicken embryos were used to isolate influenza virus from pharyngeal swab samples, respiratory tract swabs, or bronchial lavage fluid sent by surveillance point hospitals to the Center for Disease Control and Prevention (CDC) of Guangzhou.

eMethod 2 Excess mortality rate and its empirical confidence interval

Excess mortality rate: In previous studies on influenza death burden, the calculation of excess death mortality rate (EMR) can be divided into the following steps [1-6]:

1. Estimate the number of deaths at time t given the observed influenza virus activity (D_{1t}).
2. Estimate the number of baseline deaths at time t given that influenza virus did not circulate (D_{0t}), i.e. the influenza virus activity proxy was zero.
3. Calculate the daily excess death number with the difference between D_{1t} and D_{0t}, and divide the influenza-related excess death numbers by the corresponding population sizes N_t at time t and then multiplying 100 000.

The calculation formula for EMR at time t is as follows:

$$EMR_t = \frac{D_{1t} - D_{0t}}{N_t} \times 100\ 000$$

In our study, we refer to the calculation of backward attributable number in the previous studies using distributed lag non-linear model [7, 8] for estimating excess death number:

$$D_{1t} - D_{0t} = AN_{x,t} = \left[1 - \exp\left(-\boldsymbol{w}_{x,t}^{T}\boldsymbol{\eta}\right)\right] \cdot n_t$$

where $\boldsymbol{w}_{x,t}$ is the vector of transformed variables generating from a cross-basis for variable x with max lag of L at time t, $\boldsymbol{\eta}$ refers to the parameters vector of the cross-basis with n_t as the number of cases at time t. Previous studies have shown how to convert a basis variable into a cross-basis [7, 9].

Empirical confidence interval: It's difficult to obtain the confidence interval of the attributable number with an analytical formula, and the most straightforward approach is to calculate the empirical confidence interval with Monte Carlo simulations [7]. $\boldsymbol{\eta}^{(j)}$ was sampled from the multivariate normal distribution with mean vector being equal to point estimate $\hat{\boldsymbol{\eta}}$ and covariance matrix $V(\hat{\boldsymbol{\eta}})$ derived from the regression model. And then the distribution of excess death (i.e. backward attributable number) would be reconstructed empirically. An 95% empirical confidence interval (eCI) could be defined with the 2.5th and 97.5th percentiles of such distributions. In our study, for example, 5000 sets of $\boldsymbol{\eta}^{(j)}$, where $j = 1,2,\dots,5000$ were sampled from the multivariate normal distribution, calculating 5000 sets of daily excess deaths. And 5000 annual EMRs were calculated by dividing the sum of each set of daily excess deaths by the corresponding average population sizes and then multiplying 100 000. The 2.5th and 97.5th percentiles (i.e. the 125th and 4875th EMRs from the smallest to the largest) of these 5000 annual EMRs were defined as upper and lower bound of 95% eCI.

Although we had controlled the time trend and seasonality in the model, residuals still had certain autocorrelation for death from some reasons among some sex or age groups, which needs to be accounted for to obtain the correct standard error and thus the correct P value and confidence interval [10]. And we fitted the regression model as we depicted in the part of data analysis. For models that still had autocorrelation, we determined the order of autocorrelation according to PACF (eFigure 1) and used the Newey-West method to adjust the standard errors [10, 11]. Newey-West standard errors can be obtained using the *NeweyWest* function in R (package: *sandwich*) [12]. Based on the Newey-West standard error, $V'(\hat{\boldsymbol{\eta}})$ was estimated in place of $V(\hat{\boldsymbol{\eta}})$ in order to obtain new 95% eCI accounting for autocorrelation.

eMethod 3 Confidence interval of male to female rates ratios

In this study, we calculated the male to female rates ratios (MFRs) to figure out whether there is a sex difference in influenza-related excess mortality. MFRs were estimated by dividing the male excess mortality rates by the female excess mortality rates. And the 95% confidence intervals (95% CI) of MFRs were calculated by the delta method [1]:

1. Estimate the male excess mortality rate per male $\hat{p}$, female excess mortality rate per female $\hat{q}$, and $\widehat{MFR} = \hat{p}/\hat{q}$ from the data.
2. Find the natural log of $\widehat{MFR}$: $\log(\widehat{MFR})$
3. To calculate the standard error of $\log(\widehat{MFR})$, we have

$$SE(\log(\widehat{MFR})) = \sqrt{\frac{(1-\hat{p})}{\hat{p}n_1} + \frac{(1-\hat{q})}{\hat{q}n_2}}$$

where n_1 refers to the average number of males, and n_2 is the average number of females.

4. Calculate the lower and upper bound of the interval on the log scale:

$$\log(\widehat{MFR}) \pm SE(\log(\widehat{MFR}))$$

5. Exponentiate.

eMethod 4 Sensitivity analysis

Based on the main model, we changed the parameter settings of the model and conducted sensitivity analysis to check the robustness of the results. *Main model*, model for daily mortality, with five cross-basis matrices for daily temperature and daily activity proxies of each influenza subtype/lineage, maximum lag 30 days, lag-response relationships as natural cubic spline with 3 knots equidistant at the log scale, as well as a natural cubic spline with 5 degrees of freedom for day of year (1–366) and dummy variables of year to control seasonality and time trend respectively. *Mod1-Mod3*, as in main model, with a different maximum lag of 14, 21, 35 days, respectively. *Mod4,* as in main model, lag-response relationships as natural cubic spline with 3 knots equidistant at the untransformed scale, respectively. *Mod5-Mod9*, as in the main model, with a natural cubic spline with 3, 4, 6, 7 and 8 degrees of freedom for day of year, respectively. *Mod10*, as in the main model, with a periodic cubic B-spline with three equidistant knots for day of year to control seasonality. *Mod11*, as in the main model, using a linear term of day (1-1456) to control time trend. *Mod12*, as in the main model, without any term to control time trend. *Mod13*, as in the main model, without separate activity proxies for influenza B virus lineages (four cross-basis matrices for temperature, influenza A(H1N1), A(H3N2) and influenza B). *Mod14*, as in the main model, without separate activity proxies per influenza type (two cross-basis matrices, one for temperature and one for all influenza types). *Mod15*, as in the main model, with linear terms for the influenza activity proxy of each influenza subtype/lineage with a seven-days (one week) lag and a natural cubic spline with 4 degrees of freedom used for the daily temperature with the same lag, instead of cross-basis matrices.

eMethod 5 Mortality displacement

Mortality displacement, also known as harvesting effect. During the early stages of an influenza outbreak, a rapid increase of deaths occurs in high-risk groups. The rate of changing from healthy to high-risk groups is slower, and the number of people in high-risk groups decreases rapidly due to rapid initial consumption, thus greatly reducing the number of deaths, which results in a negative correlation between influenza activity and death. However, with the extension of exposure time of risk factors, the number of people at high risk gradually increases, and the number of deaths also changes, and the harmful effects of influenza will appear again [13].

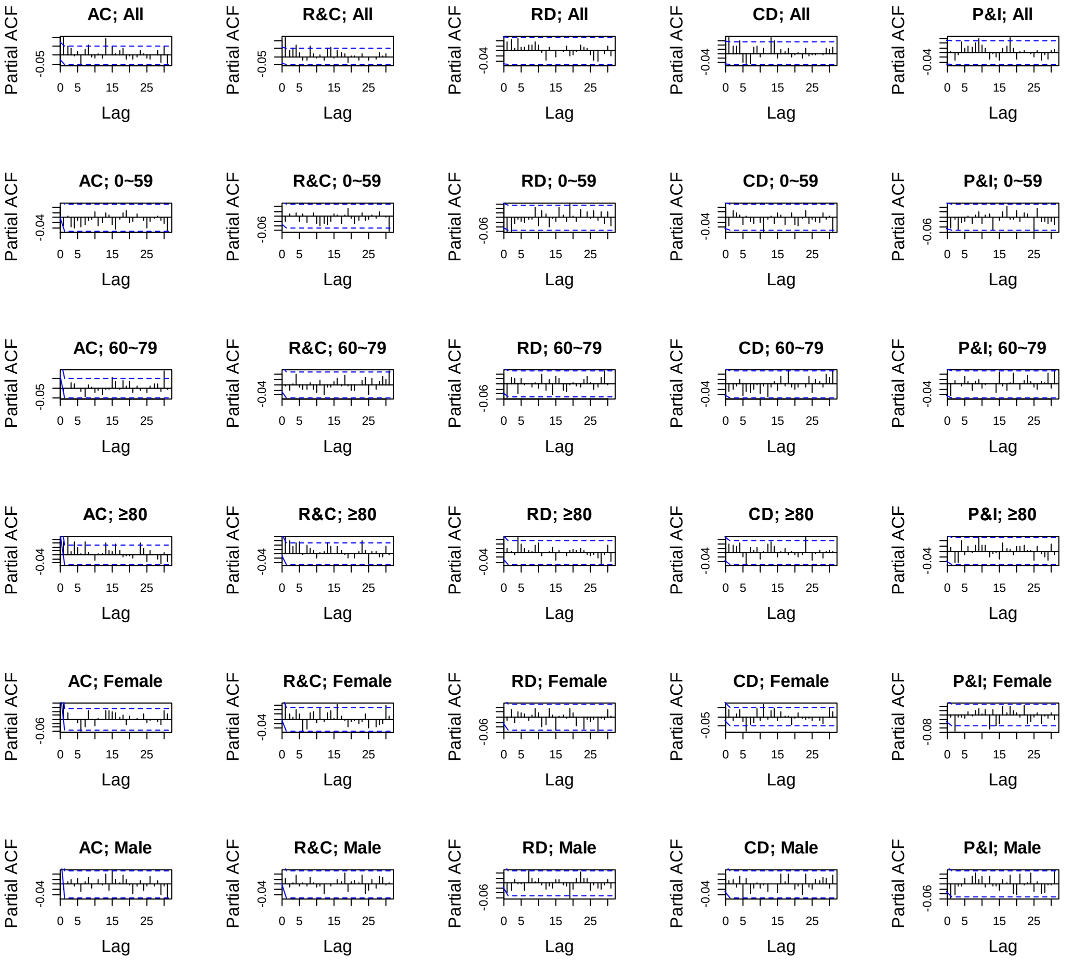

eFigure 1 Partial autocorrelation function for the residuals of models fitted for death from different reasons in population of different age groups or sex

In the study, different causes of death were modeled for the whole population (All), 0–59 years old (0~59), 60–79 years old (60~79), 80 years and older (≥ 80), and male and female. After that, partial autocorrelation coefficients (PACF) of models' residual were checked. If PACF of consecutive days starting from 1-day-lag had statistical significance, the number of consecutive days would be the order of autocorrelation, on which Newey-west method was used to correct the covariance matrix of model coefficient. Abbreviations: AC, All causes; R&C, respiratory and cardiovascular diseases; RD, respiratory diseases; P&I, pneumonia and influenza; CD, cardiovascular diseases.

References

1. Jin S, Li J, Cai R, Wang X, Gu Z, Yu H, et al. Age- and sex-specific excess mortality associated with influenza in Shanghai, China, 2010–2015. Int J Infect Dis;98:382-389. [doi:10.1016/j.ijid.2020.07.012]
2. Wu P, Goldstein E, Ho LM, Yang L, Nishiura H, Wu JT, et al. Excess Mortality Associated with Influenza a and B Virus in Hong Kong, 1998–2009. The Journal of Infectious Diseases 2012-12-15;206(12):1862-1871. [doi:10.1093/infdis/jis628]
3. Yu X, Wang C, Chen T, Zhang W, Yu H, Shu Y, et al. Excess pneumonia and influenza mortality attributable to seasonal influenza in subtropical Shanghai, China. Bmc Infect Dis;17(1). [doi:10.1186/s12879-017-2863-1]
4. Wang H, Fu C, Li K, Lu J, Chen Y, Lu E, et al. Influenza associated mortality in Southern China, 2010–2012. Vaccine;32(8):973-978. [doi:10.1016/j.vaccine.2013.12.013]
5. Li L, Liu Y, Wu P, Peng Z, Wang X, Chen T, et al. Influenza-associated excess respiratory mortality in China, 2010–15: A population-based study. The Lancet. Public health 2019-01-01;4(9):e473-e481. [doi:10.1016/S2468-2667(19)30163-X]
6. Wu S, Wei Z, Greene CM, Yang P, Su J, Song Y, et al. Mortality burden from seasonal influenza and 2009 H1N1 pandemic influenza in Beijing, China, 2007-2013. Influenza Other Resp;12(1):88-97. [doi:10.1111/irv.12515]
7. Gasparrini A, Leone M. Attributable risk from distributed lag models. Bmc Med Res Methodol 2014-01-01;14(1):55. [doi:10.1186/1471-2288-14-55]
8. Lytras T, Pantavou K, Mouratidou E, Tsiodras S. Mortality attributable to seasonal influenza in Greece, 2013 to 2017: Variation by type/subtype and age, and a possible harvesting effect. Euro surveillance : bulletin européen sur les maladies transmissibles 2019-01-01;24(14):11. [doi:10.2807/1560-7917.ES.2019.24.14.1800118]
9. Gasparrini A, Armstrong B, Kenward MG. Distributed lag non-linear models. Stat Med 2010-09-20;29(21):2224-2234. [doi:10.1002/sim.3940]
10. Bottomley C, Scott J, Isham V. Analysing interrupted time series with a control. Epidemiologic Methods;8(1). [doi:10.1515/em-2018-0010]
11. West N. A simple, positive Semi-Definite, heteroskedasticity and autocorrelation consistent covariance matrix. Econometrica;55(3):703-708. [doi: 10.2307/1913610]
12. Zeileis A. Econometric computing with HC and HAC covariance matrix estimators. J Stat Softw; 11(10):1-17. [doi:10.18637/jss.v011.i10]
13. Zanobetti A, Wand MP, Schwartz J, Ryan LM. Generalized additive distributed lag models: Quantifying mortality displacement. Biostatistics (Oxford, England) 2000-01-01;1(3):279-292. [doi:10.1093/biostatistics/1.3.279]
