# Supplementary material for: Influenza-Associated Excess Mortality by Age, Sex, and Subtype/Lineage: Population-Based Time-Series Study With a Distributed-Lag Nonlinear Model
Source: JMIR Public Health Surveill. 2023 Jan 11;9:e42530. doi: 10.2196/42530 (PMC9878364; doi:10.2196/42530)
Supplement: Multimedia Appendix 2 [file publichealth_v9i1e42530_app2.pdf]

eTable 1 Average annual excess respiratory mortality rates related to influenza by sex and age

Age group	Male		Female		MFR		
	Rate per 100 000 persons	95% eCI	Rate per 100 000 persons	95% eCI	Estimate	95% CI	P
0–59	0.28	(-0.68–0.91)	0.62	(0.15–0.85)	0.45	(0.23–0.89)	0.021
60–79	37.88	(28.45–45.72)	12.51	(7.08–16.64)	3.03	(2.75–3.33)	<0.001
≥80	280.48	(198.58–354.58)	192.98	(140.5–238.92)	1.45	(1.41–1.49)	<0.001
All ages	10.75	(8.25–13)	8.03	(6.02–9.8)	1.34	(1.17–1.54)	<0.001

Abbreviation: 95% eCI, 95% empirical confidence interval.
