# Supplementary material for: Influenza-Associated Excess Mortality by Age, Sex, and Subtype/Lineage: Population-Based Time-Series Study With a Distributed-Lag Nonlinear Model
Source: JMIR Public Health Surveill. 2023 Jan 11;9:e42530. doi: 10.2196/42530 (PMC9878364; doi:10.2196/42530)
Supplement: Multimedia Appendix 3 [file publichealth_v9i1e42530_app3.pdf]

eTable 2 Average annual excess cardiovascular mortality rates related to influenza by sex and age

Age group	Male		Female		MFR		
	Rate per 100 000 persons	95% eCI	Rate per 100 000 persons	95% eCI	Estimate	95% CI	P
0–59	1.48	(-0.35–3.02)	0.46	(-0.68–1.25)	3.24	(1.96–5.34)	<0.001
60–79	41.99	(24.9–57.82)	20.45	(8.43–31.50)	2.05	(1.90–2.22)	<0.001
≥80	236.51	(129.98–337.14)	361.21	(268.71–448.20)	0.65	(0.64–0.67)	<0.001
All ages	8.94	(5.16–12.59)	12.44	(8.59–16.14)	0.72	(0.63–0.82)	<0.001

Abbreviation: 95% eCI, 95% empirical confidence interval.
