# Supplementary material for: Influenza-Associated Excess Mortality by Age, Sex, and Subtype/Lineage: Population-Based Time-Series Study With a Distributed-Lag Nonlinear Model
Source: JMIR Public Health Surveill. 2023 Jan 11;9:e42530. doi: 10.2196/42530 (PMC9878364; doi:10.2196/42530)
Supplement: Multimedia Appendix 4 [file publichealth_v9i1e42530_app4.pdf]

eTable 3 Comparison of average annual excess respiratory and cardiovascular mortality rates attributable to all influenza estimated from different models in the sensitivity analysis.

Model	Rate per 100 000 persons	95% eCI
Main Model	20.36	(16.75–23.74)
Mod1	20.03	(16.95–22.90)
Mod2	19.22	(15.94–22.37)
Mod3	20.26	(16.30–23.70)
Mod4	19.45	(15.95–22.69)
Mod5	19.85	(16.33–23.05)
Mod6	20.01	(16.37–23.38)
Mod7	20.21	(16.59–23.53)
Mod8	19.84	(16.12–23.39)
Mod9	19.71	(15.99–23.40)
Mod10	19.17	(15.46–22.74)
Mod11	20.19	(16.52–23.53)
Mod12	20.45	(16.93–23.84)
Mod13	21.84	(18.83–24.62)
Mod14	18.98	(15.87–22.04)
Mod15	19.78	(16.82–22.51)

Abbreviation: 95% eCI, 95% empirical confidence interval.

Main model, the model we used as the principal analysis method in article. Mod1-Mod3, as in main model, with a different maximum lag of 14, 21, 35 days, respectively. Mod4, as in main model, lag-response relationships as natural cubic spline with 3 knots equidistant at the untransformed scale, respectively. Mod5-Mod9, as in the main model, with a natural cubic spline with 3, 4, 6, 7 and 8 degrees of freedom for day of year, respectively. Mod10, as in the main model, with a periodic cubic B-spline with three equidistant knots for day of year to control seasonality. Mod11, as in the main model, using a linear term of day (1-1456) to control time trend. Mod12, as in the main model, without any term to control time trend. Mod13, as in the main model, without separate activity proxies for influenza B virus lineages (four cross-basis matrices for temperature, influenza A(H1N1), A(H3N2) and influenza B). Mod14, as in the main model, without separate activity proxies per influenza type (two cross-basis matrices, one for temperature and one for all influenza types). Mod15, as in the main model, with linear terms for the influenza activity proxy of each influenza subtype/lineage with a seven-days (one week) lag and a natural cubic spline with 4 degrees of freedom used for the daily temperature with the same lag, instead of cross-basis matrices.
